# Subcutaneous Chest Wall Metastasis From Bladder Carcinoma Over the Totally Implantable Venous Access Port: A Rare Complication

**DOI:** 10.7759/cureus.70598

**Published:** 2024-10-01

**Authors:** Sotirios Botaitis, Charilaos Stamos, Sempachedin Perente

**Affiliations:** 1 First General Surgery Department, University Hospital of Alexandroupolis, Alexandroupolis, GRC; 2 Pathology Department, University Hospital of Alexandroupolis, Alexandroupolis, GRC

**Keywords:** bladder tumor, chest wall metastasis, port catheter, squamous cell carcinoma, totally implantable venous access port

## Abstract

A totally implantable venous access port (TIVAP) plays a main role in delivering chemotherapy to patients with cancer. A 70-year-old woman with bladder carcinoma presented with a mass over a port chamber of a TIVAP site. CT studies showed a mass surrounding the catheter near the port reservoir attached to the chest wall. The port was removed and the mass was biopsied. The pathology from the biopsy showed squamous cell carcinoma (SCC) from bladder carcinoma. We present a rare occurrence of chest wall metastasis at the site of a venous access port from primary bladder carcinoma.

## Introduction

Introduced in 1982 [1], totally implantable port systems have been increasingly utilized across various medical specialties to facilitate vascular access, especially in oncology patients. However, there are complications associated with these devices. The overall complication rate has been reported to be 7.2-12.5%, with port system infection being the most common one [2]. Other complications affiliated with totally implantable venous access ports (TIVAPs) are hemorrhage, pneumothorax, hemothorax, malpositioning, catheter rupture, and venous thrombosis [3]. The development of metastasis at the catheter entry points and especially over the port chamber is an extremely rare complication, although there are some case reports in the literature. We present a case of subcutaneous metastasis localized to the port chamber on the chest wall in a patient with bladder carcinoma, following the placement of a TIVAP.

## Case presentation

A 70-year-old woman with a medical history of bladder carcinoma with stage IV (T4N3M1) presented to the Department of General Surgery for the implantation of a TIVAP to facilitate chemotherapy administration. The patient underwent only a biopsy of the primary tumor, and during the placement of the port, she had metastases to the lungs and iliac lymph nodes. A TIVAP (BardPort, M.R.I. Hard Base Implantable Port with Attachable 8-F Groshong Single Lumen Venous Catheter, Salt Lake City, USA) was placed in the right internal jugular vein using the Seldinger technique.

The patient was placed on the surgical bed, and a right internal jugular vein approach was chosen; then she was maintained in the Trendelenburg position to decrease the risk of air embolism and fill up the internal jugular vein. Under local anesthesia, without additional intravenous sedation, the needle was inserted over the right internal jugular vein with ultrasound guidance. After the right internal jugular vein was punctured, a flexible J guidewire was introduced through the needle. A small skin incision (0.5 cm) was made at the guidewire exit site in the neck to make the peel-away sheath easy to pass. A vein dilator and peel-away sheath were used to insert the catheter. On either side of the right clavicle, in the middle line, an incision diameter (3 cm) was made to implant the port reservoir into the chest wall. The venous port was fixed to the subcutaneous pocket above the major pectoral muscle, and the incision in the skin was sutured with individual sutures.

The patient underwent a routine post-procedure chest X-ray to rule out pneumothorax or other complications. No pneumothorax was found, and she was discharged.

**Figure 1:**
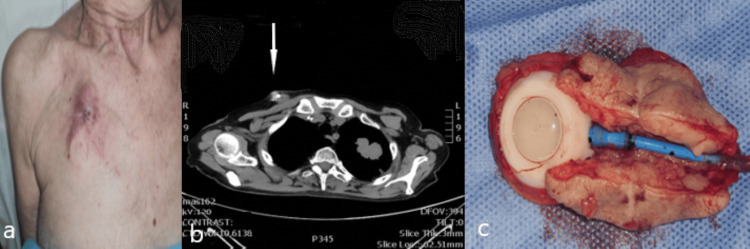
(a) Macroscopic overview of the patient; (b) CT scans showed the mass near the chamber of the port; and (c) The surgical specimen that was removed en bloc (port and mass) opening along the catheter

**Figure 2:**
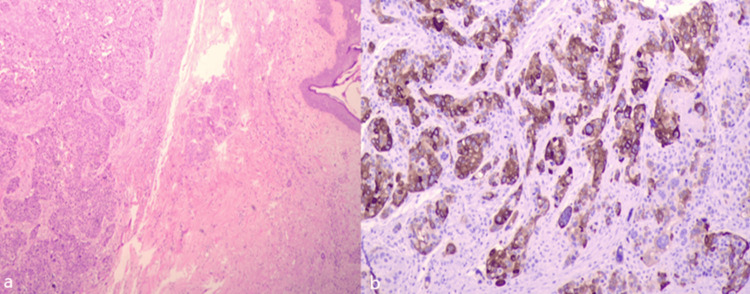
(a) Histopathological examination of the specimen (AHx4) and (b) Uroplakin (x10)

She received radiotherapy and chemotherapy regimen with gemcitabine hydrochloride (carboplatin cis-diammine(1,1-cyclobutanedicarboxylato)platinum(II)), enfortumab vedotin (Javlor), and INN-vinflunine detartrate. After nine months, when the subcutaneous metastasis occurred, she received immunotherapy with Nivolumab. She presented with swelling and erythema around the port reservoir, associated with pain and redness. She didn’t report any fever or bleeding from the port site. Physical examination revealed a hard mass with irregular borders over the port reservoir of the TIVAP on the chest wall (Figure 1a). The CT scan revealed metastases to the lungs and a mass around the catheter near the port chamber (dimensions 3 x 2 cm) (Figure 1b). Consequently, the TIVAP was removed en bloc with the mass under local anesthesia (Figure 1c). A biopsy of the mass showed invasive SCC spread from bladder carcinoma (Figures 2a-2b). The patient denied a new port device, continued the therapy from the peripheral vein, and died six months later.

## Discussion

Patients with carcinoma often require placement of TIVAPs to provide easy vascular access to facilitate the delivery of chemotherapy, especially in the outpatient setting, as this allows patients unrestricted mobility and greater freedom of activities. Even though the implantable port system seems to solve the problem of vascular access, there are complications associated with the procedure itself or the permanence of the catheter. The most extremely rare complication is the metastatic spread at the site of the TIVAP, as has been reported only six times in the literature. Two reports of tumor metastasis regarding patients with non-small cell lung cancer and mesothelioma [4,5], the other two patients with squamous cell carcinoma (SCC) of the tongue and a spindle cell carcinoma of the hard palate [6,7], one patient with SCC of the hypopharynx [6,7], and one patient with advanced gastrointestinal stromal tumor (GIST) [8]. It nominates that tumor seeding may be a potential complication of central venous catheter (CVC) installation [4].

A definite pathophysiology explanation behind tumor spread remains unclear. Metastasis is a vastly complex process involving tumor cell motility, intravasation, and circulation in the blood or lymph system, extravasation, and growth in new tissues and organs [9]. The development of metastasis around the TIVAP may be influenced by factors such as the type of cancer, the wound healing process at the implantation site, or the presence of a long-term CVC, which could lead to chronic inflammation or a combination of these factors. According to the type of cancer, the most common histopathology is SCC regarding tumor seeding at the site of a TIVAP [8]. In our case, the histological examination showed urothelial carcinoma (UC) with squamous differentiation (SD). Bladder cancer has variable metastatic potential, and almost any organ can be involved in metastasis [10]. Lymph nodes, bones, lungs, liver, and peritoneum were the most common sites of metastasis from bladder cancer. Once bladder cancer has reached the lymph nodes, it can travel to distant parts of the body through the lymphatic system [10]. UC can develop SD (UCSD), which occurs in up to 20% of UC in bladder cancer cases and is associated with advanced tumor stage [10]. The biological characteristics and clinical significance of SD of bladder carcinoma are poorly understood.

Chronic inflammation has been closely associated with cancer development. Both inflammation and cancer involve processes such as cell proliferation, survival, and migration, which are regulated by growth factors, cytokines, and inflammatory and angiogenic signals originating from the tumor and wound microenvironment [10]. Based on this, we hypothesize that the inflammatory microenvironment may have led to tumor seeding at the site of the catheter. In our patient, there was no arterial damage or pneumothorax during the surgery, and the catheter did not pass through any metastatic mass in the chest. However, the patient developed metastasis at the chest wall nine months after catheter placement. We believe that the inflammatory processes of wound healing around the trauma due to the process of TIVAPs may cause inflammation and vascularization along the port, giving rise to implantation and growth of the tumor cells along the catheter tract.

In addition, the placement of a totally implantable venous system for cancer patients must adhere to strict preventative measures. It should never be placed in direct contact with a cancerous focus. To avoid contamination, the surgical instruments used for implanting the system must be separate from those used in cancer-related procedures. Moreover, the system should not be implanted in the operating room during cancer surgery, as this increases the risk of device contamination and infection, and the location may not be ideal. For unilateral head, neck, or thoracic tumors, it is preferable to place the device on the opposite side of the tumor. Alternatively, a CVC can be inserted via the arm using a peripherally inserted central catheter (PICC port). Complete excision of metastases should be performed with clear margins, removing both the tumor and the implantable port in one procedure. To our knowledge, no prior cases of bladder carcinoma with metastasis spread to a TIVAP have been reported.

## Conclusions

The placement of CVCs, although they play an important role in the administration of chemotherapy, especially on an outpatient basis, may be associated with the occurrence of rare complications such as the development of metastases along the catheter. It is important to remember that the implantation of a totally implantable venous system must be conducted under strict conditions to prevent inflammation and cancer spread. The procedure should never be performed in proximity to a cancerous site. Even though this is an extremely rare complication, there is a possibility of metastasis developing above the totally implantable venous system, especially in a patient with aggressive and advanced-stage cancer.
